# Amniotic Membrane-Derived Stromal Cells Release Extracellular Vesicles That Favor Regeneration of Dystrophic Skeletal Muscles

**DOI:** 10.3390/ijms241512457

**Published:** 2023-08-05

**Authors:** Martina Sandonà, Federica Esposito, Anna Cargnoni, Antonietta Silini, Pietro Romele, Ornella Parolini, Valentina Saccone

**Affiliations:** 1 Istituto di Ricovero e Cura a Carattere Scientifico (IRCCS), Fondazione Santa Lucia, Via Fosso di Fiorano 64, 00143 Rome, Italy; m.sandona@hsantalucia.it (M.S.); federica.esposito@uniroma1.it (F.E.); 2 Unit of Histology and Medical Embryology, Division DAHFMO, University of Rome La Sapienza, 00185 Rome, Italy; 3 Centro di Ricerca “E. Menni”, Fondazione Poliambulanza Istituto Ospedaliero, 25124 Brescia, Italy; anna.cargnoni@poliambulanza.it (A.C.); antonietta.silini@poliambulanza.it (A.S.); pietro.romele@poliambulanza.it (P.R.); 4 Department of Life Science and Public Health, Università Cattolica del Sacro Cuore, Largo F. Vito 1, 00168 Rome, Italy; 5 Fondazione Policlinico Universitario “Agostino Gemelli” IRCCS, Largo A. Gemelli, 00168 Rome, Italy

**Keywords:** amnion, extracellular vesicles, Duchenne muscular dystrophy, mesenchymal stem/stromal cells, skeletal muscle regeneration

## Abstract

Duchenne muscular dystrophy (DMD) is a muscle disease caused by mutations in the dystrophin gene characterized by myofiber fragility and progressive muscle degeneration. The genetic defect results in a reduced number of self-renewing muscle stem cells (MuSCs) and an impairment of their activation and differentiation, which lead to the exhaustion of skeletal muscle regeneration potential and muscle replacement by fibrotic and fatty tissue. In this study, we focused on an unexplored strategy to improve MuSC function and to preserve their niche based on the regenerative properties of mesenchymal stromal cells from the amniotic membrane (hAMSCs), that are multipotent cells recognized to have a role in tissue repair in different disease models. We demonstrate that the hAMSC secretome (CM hAMSC) and extracellular vesicles (EVs) isolated thereof directly stimulate the in vitro proliferation and differentiation of human myoblasts and mouse MuSC from dystrophic muscles. Furthermore, we demonstrate that hAMSC secreted factors modulate the muscle stem cell niche in dystrophic–*mdx*-mice. Interestingly, local injection of EV hAMSC in *mdx* muscles correlated with an increase in the number of activated Pax7+/Ki67+ MuSCs and in new fiber formation. EV hAMSCs also significantly reduced muscle collagen deposition, thus counteracting fibrosis and MuSCs exhaustion, two hallmarks of DMD. Herein for the first time we demonstrate that CM hAMSC and EVs derived thereof promote muscle regeneration by supporting proliferation and differentiation of resident muscle stem cells. These results pave the way for the development of a novel treatment to counteract DMD progression by reducing fibrosis and enhancing myogenesis in dystrophic muscles.

## 1. Introduction

Mesenchymal stromal cells (MSCs) have become increasingly attractive for neurodegenerative disorders [1]. In these recent years, numerous studies on mesenchymal stromal cells (MSCs) have elucidated and highlighted their role in tissue repair in different disease models [2,3,4]. Their suitability is ascribed to a paracrine effect rather than to a direct differentiation [5,6,7]. MSCs work synergistically with the endogenous microenvironment to stimulate resident cell proliferation and enhance the regenerative potential in injured tissue. In particular, recent research on extracellular vesicles (EVs) released by MSCs, including exosomes, has grown significantly. EVs mediate the transport of genetic materials and signaling factors as a novel mode of communication between cells. It is copiously described that EVs contribute to several important physiological functions such as immune responses, tissue repair, and neural communication via their role(s) in intercellular transport [8]. Among MSCs, perinatal MSC, and in particular those derived from the amniotic membrane (hAMSCs), have conspicuous features for applications in regenerative medicine, such as their ability to modulate the immune response [9,10,11,12].

Transplantation of hAMSCs has repeatedly been shown to favor tissue repair and regeneration in rodent models of inflammatory-based diseases [13,14,15,16]. Their great beneficial characteristics represent an authentic elixir that is constituted by a promising source of factors, cytokines, and EVs useful for regenerative medicine.

Skeletal muscle is a very adaptive tissue, but its regenerative potential is altered during acute damage and chronic genetic conditions. Recent works demonstrate that both MSCs and their secretome are able to help myofiber regeneration and enhance myogenesis and, interestingly, can be exploited as a novel strategy for therapeutic interventions in muscular diseases such as muscular dystrophies or atrophy [17,18,19,20,21].

Skeletal muscle is composed of single myofibers that represent the complexity of the muscular stem cell niche in which several cell types cooperate to regulate the balance between quiescence/activation/differentiation of the resident muscle stem cells (MuSCs), and to support muscle regeneration [22,23].

The myogenic program that is activated during acute muscle damage appears compromised and deregulated during chronic diseases such as in Duchenne muscular dystrophy (DMD), where inflammatory and muscle resident cells lose their ability to modulate repair and regeneration [24].

DMD is among the most severe and incurable form of genetic diseases. It is caused by mutations in the *dystrophin* gene and it affects the skeletal muscle that is characterized by myofiber fragility and successive cycles of muscle necrosis and regeneration whereby muscle is replaced by fibrotic and fatty tissue [25].

Skeletal muscle regeneration depends on adult muscle stem cells (MuSCs) and chronic inflammation impairs MuSC activation and differentiation, leading to insufficient skeletal muscle regeneration that exhausts during DMD progression.

Hence, strategies to improve MuSC function and preserve muscle stem cell niche represent a promising avenue to extend the regenerative potential in neuromuscular disorders.

EVs have been shown to play important functions in the normal maintenance and degeneration of musculoskeletal tissues. It has been demonstrated that EVs play a role in muscle physiological processes such as growth and development [26], muscle regeneration following injury or chronic illness [27,28,29,30], and also in muscle wasting and aging [31].

Indeed, we have recently highlighted a role for EV-derived miRNAs isolated from fibroadipogenic progenitors (FAPs) in Duchenne muscular dystrophy [30].

Different sources of MSCs have been studied for their therapeutic potential to exert some beneficial effects in mouse and animal DMD models, however, the effects of amniotic membrane MSCs were never reported for DMD [21].

Herein, we aimed to determine if hAMSC secreted factors, including EVs, could favor skeletal muscle regeneration by modulating the MuSC niche, and more specifically have an effect on progenitor cell proliferation and differentiation. Our results from the studies present in literature and above-mentioned demonstrate that the EVs isolated from hAMSCs can provide a new therapeutic approach based on the delivery of bioactive particles into dystrophic muscles that counteract the rapid development of disease, and in part, restore an environment that in dystrophic muscles is seriously compromised without causing the unwanted systemic side effects of cell therapies.

## 2. Results

### 2.1. Conditioned Medium from Amniotic Cells Activates Both Human and Mice Dystrophic Myoblasts

**Figure 1:**
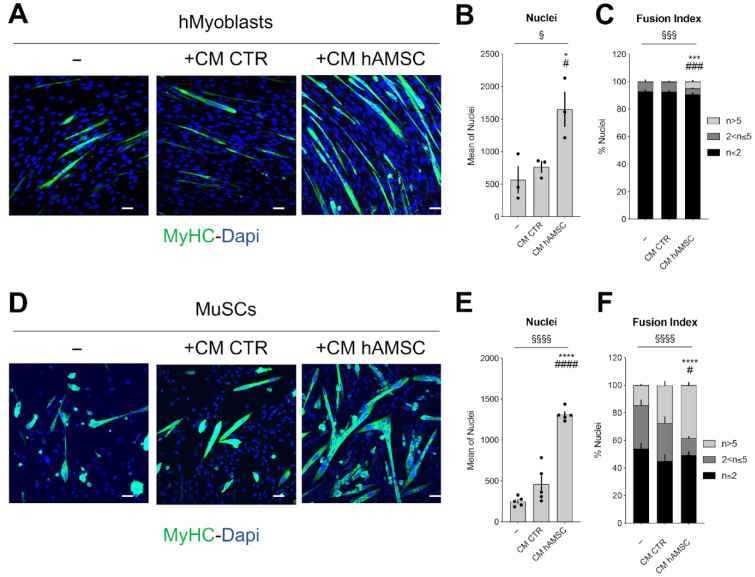
CM hAMSC improves the proliferation and differentiation in dystrophic human myoblasts and in dystrophic MuSCs. (**A**) Representative images showing the myogenic differentiation of dystrophic human myoblasts cultured either alone (−) or with CM hAMSC (+CM hAMSC) or CM CTR (+CM CTR). Myogenic differentiation was assessed by immunostaining for MyHC (green). Nuclei were counterstained with DAPI (blue); Scale bar = 50 µm. (**B**) Graph showing the mean nuclei number in the condition described in (**A**) (n = 3, biological replicates). (**C**) Graph showing the fusion index of human myoblasts in the condition described in (**A**) (n = 3, biological replicates). (**D**) Representative images showing the myogenic differentiation of dystrophic MuSCs cultured either alone (−) or with CM hAMSC (+CM hAMSC) or CM CTR (+CM CTR). Myogenic differentiation was assessed by immunostaining for MyHC (green). Nuclei were counterstained with DAPI (blue); Scale bar = 50 µm. (**E**) Graph showing the mean nuclei number in the condition described in (**D**) (n = 5, biological replicates). (**F**) Graph showing the fusion index of dystrophic MuSCs cultured in the conditions described in (**D**) (n = 5, biological replicates). All data correspond to the average ± SEM. § Means statistical analysis by one-way or two-way ANOVA test; ^§^ *p* < 0.05, ^§§§^ *p* < 0.001, ^§§§§^ *p* < 0.0001. Star (*) indicates statistical analysis by Tukey’s test relative to human myoblast or MuSCs cultured alone (−); * *p* < 0.05, *** *p* < 0.001, **** *p* < 0.0001. Hashtag (#) means statistical analysis by Tukey’s test relative to human myoblast or MuSCs cultured CM CTR; ^#^ *p* < 0.05, ^###^ *p* < 0.001, ^####^ *p* < 0.0001.

The conditioned medium from human amniotic cells (CM hAMSC), that represents the secretome of amniotic mesenchymal stromal cells, revealed a novel ability to support proliferation and consequently differentiation of dystrophic human myoblasts showing partial recovery of the compromised activity of cells isolated from biopsies of DMD patients. As shown in Figure 1A, DMD patient-derived human myoblasts (hMyoblasts) proliferate more and can form bigger myotubes when cultured in the presence of CM hAMSC (+CM hAMSC) if compared to myoblasts cultured in the presence of CTR-CM (+CM CTR) or alone (−) (Figure 1A–C). These data indicate that the secretome of hAMSC enhanced the ability of dystrophic myoblasts to differentiate into multinucleated myotubes. Based on these encouraging results we decided to investigate the regenerative potential of CM hAMSC and to do this, we moved to a suitable mouse DMD disease model–*mdx* mice. Muscle stem cells (MuSCs) were isolated from 1.5 month-old *mdx* mice and cultured either in growth medium, GM2 (−), or in GM2 with non-conditioned control medium—CM CTR (+CM CTR) or with CM hAMSC (+CM hAMSC). After 48 h, the proliferation and myogenic differentiation of MuSCs were evaluated. When compared to MuSCs cultured in GM2 (−), those cultured in the presence of CM hAMSC expanded and there was a 20% increase of MuSCs fusion index (Figure 1D–F). There was also a small and not significant effect in differentiation also for MuSCs cultured in CM CTR. These data, in combination with the effects on human samples, confirmed the ability of the hAMSC secretome to influence MuSCs function.

### 2.2. hAMSC Conditioned Medium Promotes MuSCs Activation and Proliferation

**Figure 2:**
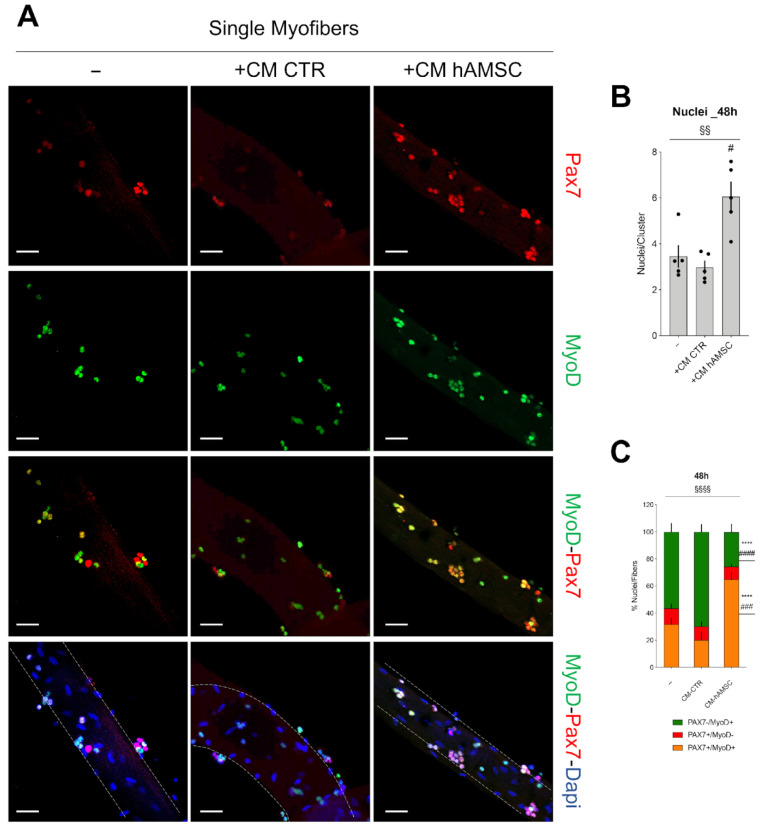
CM hAMSC improves MuSCs activation and proliferation. (**A**) Representative images of myofibers cultured for 48 h alone (−) or with CM hAMSC (+CM hAMSC) or CM CTR (+CM CTR). Myofibers were stained with anti-Pax7 (Red) and anti-MyoD (Green). Nuclei were counterstained with DAPI (blue). Scale bar = 50 µm. (**B**) The graph shows the average nuclei present under the fiber-niche (cluster) in the condition described in (**A**). (n = 5, biological replicates). (**C**) The graph shows the percentage of nuclei present under the fiber-niche (cluster) that are positive for PAX7 (Pax7+/MyoD−), for MyoD (PAX7−/MyoD+), or both (PAX7+/MyoD+) in the condition described in (**A**), (n = 5, biological replicates). All data correspond to the average ± SEM. § Means statistical analysis by one-way and two-way ANOVA test; ^§§^ *p* < 0.01; ^§§§§^ *p* < 0.0001. Star (*) indicates statistical analysis by Tukey’s test relative to myofibers cultured alone (−); **** *p* < 0.0001. Hashtag (#) means statistical analysis by Tukey’s test relative to myofibers cultured with CM CTR; ^#^ *p* < 0.05; ^###^ *p* < 0.001; ^####^ *p* < 0.0001.

The increased number of cells observed in Figure 1 led us to hypothesize an expansion of MuSCs after exposure to the entire supernatant derived from hAMSCs culture. To investigate this hypothesis, we evaluated the effect of the hAMSC secretome (CM hAMSC) on MuSCs within single fibers isolated from WT mice. Freshly isolated single muscle fibers were incubated with CM hAMSC and the effect on MuSCs was evaluated by monitoring the expression of Pax7 and MyoD after 48 h from the incubation compared to the effect on MuSCs grown either in GM1 medium alone (−) or in GM1 added with CM CTR. Compared to control samples, single fibers cultured with CM hAMSC doubled in number of nuclei present under the fiber-niche in the cluster, highlighting an expansion of satellite cell pools (Figure 2A,B). In addition, the exposure of single myofibers with CM hAMSC caused a clear reduction in the number of committed MuSCs (Pax7-/MyoD+) and a strong increase by 50% of the number of Pax7+/MyoD+ MuSCs compared to control conditions (either in absence of CM hAMSC and in the presence of CM CTR) considered to be activated MuSCs (Figure 2A,C). These results indicate the ability of the CM hAMSC to promote expansion and activation of the Pax7+/MyoD+ MuSCs, opening the way to hypothesize a therapeutic role in DMD therapy that is characterized by a depauperation of the muscle stem cells reservoir. 

### 2.3. Extracellular Vesicles with Exosome Features Mediate the Ability of hAMSC to Promote MuSC Activation and Differentiation

As indicated for other MSCs, structural analyses revealed that hAMSCs actively release extracellular vesicles (EVs) which can transfer genetic material to target cells [32]. In this study, we investigated whether the mediator of the myo-regenerative potential of the CM hAMSC could be associated to their EVs. Size distribution analysis of EVs isolated from CM hAMSC (EVs hAMSC) confirmed features recently published by Ragni et al. [32], and more specifically a standard dimension profile of approximately 100 nm [33] and a round shape as revealed by SEM analyses (Figure S1A–C). Finally, Western blot analysis showed that exosome markers including the tetraspanin CD81 and Alix were expressed in EVs hAMSC. It is to be underlined that no EVs were able to be isolated from the CTR medium (Figure S1D). 

To investigate if the vesicular component isolated from CM hAMSC could mediate MuSC activity, dystrophic MuSCs were cultured in the presence of CM hAMSC previously treated with the neutral sphingomyelinase inhibitor GW4869 to selectively block exosome biogenesis or with respective vehicle (DMSO), as described in the Section 4 [34,35].

**Figure 3:**
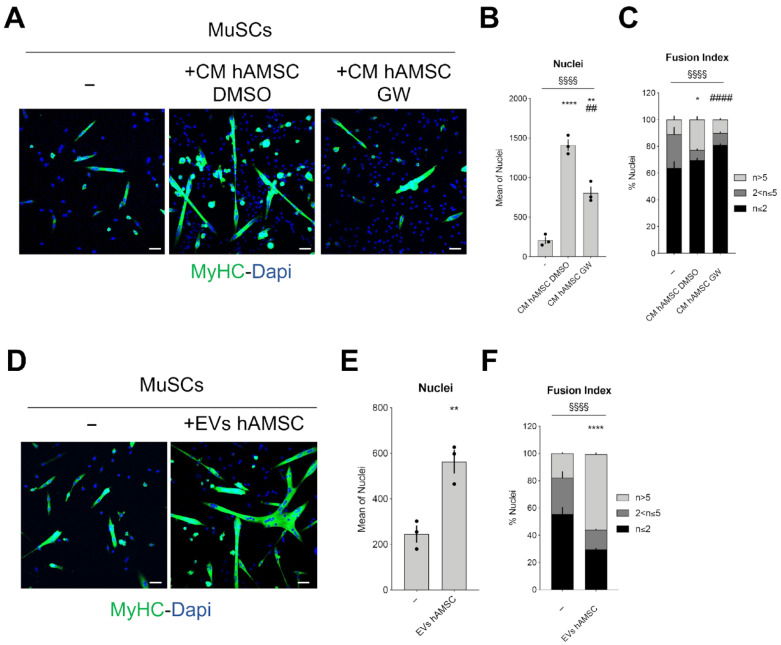
EVs hAMSC are responsible for the beneficial effects exerted by CM hAMSC on MuSC differentiation. (**A**) Representative images showing the myogenic differentiation of dystrophic MuSCs cultured either alone (−) or with CM hAMSC exposed to DMSO or GW4869 (+CM hAMSC DMSO; +CM hAMSC GW). Myogenic differentiation was assessed by immunostaining for MyHC (green). Nuclei were counterstained with DAPI (blue); Scale bar = 50 µm. (**B**) Graph showing the mean nuclei number in the condition described in (**A**) (n = 3, biological replicates). (**C**) Graph showing the fusion index of dystrophic MuSCs cultured in the conditions in (**A**) (n = 3, biological replicates). All data in (**B**,**C**) correspond to the average ± SEM. § Means statistical analysis by two-way ANOVA test; ^§§§§^ *p* < 0.0001. Asterisk (*) indicates statistical analysis by Tukey’s test relative to MuSCs cultured alone (−); * *p* < 0.05; ** *p* < 0.01; **** *p* < 0.0001. Hash (#) indicates statistical analysis by Tukey’s test relative to MuSCs cultured with CM hAMSC DMSO; ^##^ *p* < 0.01; ^####^ *p* < 0.0001. (**D**) Representative images showing the myogenic differentiation of dystrophic MuSCs cultured either alone (−) or with the EVs isolated from the conditioned media of hAMSC (+EV hAMSC). Myogenic differentiation was assessed by immunostaining for MyHC (green). Nuclei were counterstained with DAPI (blue); Scale bar = 50 µm. (**E**) Graph showing the mean nuclei number in the condition described before. (n= 3, biological replicates). All data correspond to the average ± SEM. Star (*) indicates statistical analysis by unpaired *t*-test; ** *p* < 0.01. (**F**) Graph showing the fusion index of dystrophic MuSCs cultured in the conditions described before (n = 3, biological replicates). All data correspond to the average ± SEM. § Means statistical analysis by two-way ANOVA test; ^§§§§^ *p* < 0.0001. Asterisk (*) indicates statistical analysis by Tukey’s test relative to MuSCs cultured alone (−); **** *p* < 0.0001.

The exposure to GW4869 considerably decreased the ability of CM hAMSC to enhance MuSC activity; the number of nuclei were reduced by approximately half and there was a significant reduction of satellite differentiation into myotubes (Figure 3A–C). 

Then, to confirm whether hAMSC-derived EVs could deliver regenerative messages to dystrophic MuSCs, MuSCs were incubated with EVs isolated from the serum-free conditioned medium of hAMSCs for 48 h. When compared to MuSCs alone (−), those cultured in hAMSC-derived EVs had a tripled number of cell nuclei and increased by more than 30% their ability to form multinucleated myotubes (Figure 3D–F). In comparison to the results shown in Figure 3 obtained using purified EVs, the results relative to CM hAMSC (Figure 1 and Figure 2) clearly demonstrate that the whole secretome has a prominent role on the proliferation of MuSCs, probably by delaying the differentiation process, and the derived vesicular component mainly supports MuSC differentiation (Figure S1E,F). All these indications imply that EVs are able to contribute to MuSC expansion, activity, and positively influence MuSC biological properties that in a dystrophic environment are altered by degenerative stimuli. 

### 2.4. EVs Released by hAMSCs Promote Regeneration and Reduce Fibrosis in Dystrophic Muscles

We then explored the potential of hAMSC-released EVs to support MuSC regeneration in vivo within the pathologic context of the dystrophic mice muscles. 

We investigated the functional impact on muscle regeneration by monitoring the effect of EVs hAMSC injection on parameters of disease progression in 1.5-month old *mdx* mice. The age of treatment was set at 1.5 months since this reflects the regenerative window for *mdx* mice and because it is well known that in this timeframe satellite cells are not exhausted. In particular, there are a great number of studies that demonstrate that the environment is still permissive for regeneration in 6–8 week-old *mdx* mice, at which timepoint the muscle is not completely compromised by fibrotic scars [30,36,37,38]

**Figure 4:**
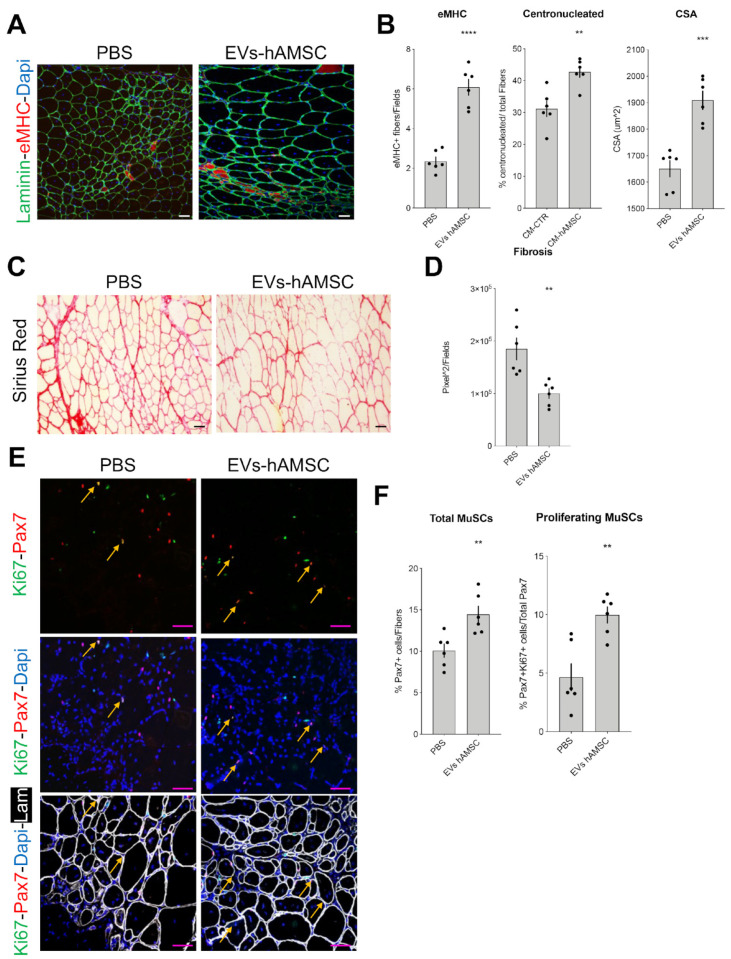
EVs hAMSC counteract dystrophic muscle degeneration and improve regeneration by reducing fibrosis and expanding MuSCs. (**A**–**F**) Stainings and relative measurements on tibialis anterior muscle transversal sections of 1.5-month-old *mdx* mice treated once a week for 21 days with intramuscular injections (tibialis anterior) of PBS (PBS), or EVs derived from conditioned media of hAMSC (EVs hAMSC). (n = 6, biological replicates). Asterisk (*) indicates statistical analysis by unpaired *t*-test; ** *p* < 0.01, *** *p* < 0.001, **** *p* < 0.0001. Nuclei were counterstained with DAPI (blue). All data correspond to the average ± SEM. (**A**) Representative images of immunofluorescence for embryonic myosin heavy chain (eMyHC—red) and laminin (laminin—cyan) stainings. Scale bar = 50 µm. (**B**) The graph on the left shows the quantification of muscle regeneration (eMyHC); the graph in the middle shows the percentage of centrally nucleated fibers and the graph on the right shows the quantification of cross-sectional area (CSA). (**C**) Representative images of Sirius red staining. Scale bar = 50 µm. (**D**) Graph showing the quantifications of fibrotic area. (**E**) Representative images of Pax7 (Red), Ki67 (Green), and laminin (Lam-White) stainings. Arrows indicate single and double positive cells for Pax7, Ki67. Scale bar = 50 µm. (**F**) The graph on the left panel shows the quantifications total MuSCs considering the percentage of Pax7-positive cells relative to the number of the fibers. The graph on the right shows the quantifications of proliferating MuSCs, considering the percentage of Pax7+Ki67+ cells with respect of the total amount of Pax7+ cells.

Tibialis anterior (TA) of *mdx* mice were injected with 20 µL of EVs hAMSC, corresponding to 3.2 × 10^9^ vesicles, every 7 days for 3 times. According to our previous work, the animals were sacrificed 7 days after the last injection so that there were no influences due to administration injury, then the harvested muscles were fully analyzed [30] (Figure S2A). We compared the ability of EVs purified from CM hAMSC to stimulate regeneration and reduce fibrosis compared to PBS-injected mice. Analysis of the muscle sections of whole muscles treated with EVs hAMSC resulted in an increase in new fiber formation, as shown by embryonic-MyHC-positive myofibers (eMyHC) and centronucleated fiber counts; moreover, an increase of cross-sectional area (CSA) was evident, suggesting the ability of EVs hAMSC to counteract dystrophic muscle degeneration (Figure 4A,B). In addition, EVs hAMSC were also able to reduce fibrotic tissue deposition and scar formation in *mdx* mice (Figure 4C,D). Finally, when compared to muscles from vehicle-treated *mdx* mice, EVs hAMSC induced an increase in Pax7-positive cells (~5%), predominantly located in the sub-laminar position within muscles from *mdx* mice, and in particular Pax7-Ki67-positive cells that represent the proliferating MuSCs (Figure 4E,F). Given that Pax7 is a typical marker of MuSCs [36,37], the increased number of proliferating Pax7-positive cells in *mdx* muscles exposed to EVs hAMSC indicates that EVs are packaged with a cargo that regulates MuSC number and Pax7 expression in regenerating muscles (Figure 4E,F). Molecular analysis on the transcripts of the main genes involved in myogenesis: Pax7 and MyoG, confirmed histological results showing an increase in *Pax7* and *Myogenin* gene expression in muscles after EVs hAMSC injection. On the other hand, the same transplanted muscles show a reduction in *Col1a1* and *Col3a1* that are the main actors of fibrosis, supporting the in vivo results on EVs hAMSC activity on degenerative hallmarks of DMD (Figure S2B). 

## 3. Discussion

In this study, we revealed the potential of hAMSC secretome and the EV fraction derived thereof in mediating a proliferative and differentiative effect on dystrophic muscles. 

Previous studies demonstrated the ability of hAMSC to promote tissue regenerative processes through paracrine mechanisms in inflammation-based diseases [15]. hAMSC and their secreted bioactive molecules have been shown to favor the resolution of injury mainly through the modulation of innate and adaptive immunity, resulting in an effective approach for chronic inflammatory disorders, autoimmune diseases, and non-healing wounds [16,38,39].

In this study, we evidenced the never-described feature of the hAMSC secretome to promote dystrophic muscle regeneration independently of its well-known immunomodulatory activity. Specifically, we demonstrated the ability of the hAMSC secretome to act directly on the MuSC niche by supporting progenitor cell proliferation and differentiation that are widely impaired in DMD muscles. Considering that, similar to other types of MSCs [40,41], the effects of the hAMSC secretome can be mediated by bioactive factors released as free molecules and also conveyed by extracellular vesicles (EVs) [32], and taking into account that our previous data demonstrated that EVs secreted by muscle-resident MSCs (i.e., fibro-adipogenic progenitors) play an important role in the modulation of dystrophic muscle regeneration [30], we explored the effects exerted by EVs hAMSC. 

Inhibiting in hAMSC the EVs release using the neutral sphingomyelinase inhibitor, we observed a possible differential role in muscle regeneration for the whole hAMSC secretome (CM hAMSC) and the EV fraction derived thereof. Our data indeed highlight a predominant role of freely secreted factors in shaping the proliferative ability of MuSCs (higher increment in the total number of nuclei upon CM hAMSC treatment) while EVs mostly govern the differentiation ability of MuSCs (higher fusion index in the presence of EVs hAMSC). 

Since it can be speculated that the major pro-proliferative effect of CM hAMSC could be attributed to a possible delay in MuSC differentiation, we chose to focus on the potential of EVs hAMSC, that not only maintained a pro-proliferative effect but also induced differentiation, to support MuSCs regeneration in vivo in dystrophic mouse muscles. 

Another important finding of this study is that a local treatment with EV hAMSC can significantly support in vivo muscle regeneration in dystrophic mice. Indeed, we observed reduced levels of muscle fibrosis, a condition known to negatively affect muscle regeneration, contributing to a severe impairment of muscle function. Interestingly, we also observed an increased formation of new myofibers, increased myofiber size, and number of proliferating MuSCs in myofibers. These results are in agreement with those obtained in other studies exploring the potential therapeutic efficacy of EVs derived from fetal perinatal cells other than hAMSCs on muscle regeneration. Two independent studies indeed observed that secretome, and in particular EVs from amniotic fluid stem cells (hAFS), dampen muscle inflammation, counteracting the development of necrotic muscle fibers in a mouse model of muscular atrophy (HSA-Cre, Smn^F7/F7^ mice) [41] and are able to enhance proliferation, and protect against cellular senescence in the CTX mouse model [42]. Another study found that EVs from Wharton’s Jelly MSC (hWJ MSC) down-regulate the inflammatory environment and increase the levels of MHC3+ cells in injured muscles of mice subjected to surgical muscle removal [20]. 

The only study showing a direct effect of conditioned medium and exosomes derived from placental MSC reported that, similar to our findings, treatment increased the differentiation of myoblasts from Duchenne patients and *mdx* mice and reduced the expression of fibrogenic genes in DMD patient myoblasts. However, differently from the amniotic MSC used in this study, derived from a specific and characterized placental tissue of fetal origin (the amniotic membrane), the specific tissue source cannot be defined for placental MSC. Indeed, placental tissues are heterogeneous tissues including the placental disc, umbilical cord, amniotic fluid, and amniotic sac [18]. 

These studies evidence that the regenerative action of EVs is the result of multiple effects, such as anti-inflammatory, pro-angiogenic, anti-apoptotic, and anti-fibrotic ones, however, none of these investigated the effect of EVs directly on freshly isolated fibers from murine skeletal muscle, allowing study of MuSCs activation and proliferation in the muscle compartment/muscle niche. 

The open question remains as to which factors mediate the regenerative effects of the hAMSC secretome on muscle. Based on our previous study which identified molecules and miRNA present in the hAMSC secretome [32], and taking advantage of other studies in this area, several factors in CM hAMSC can be potentially linked to its therapeutic role in dystrophic muscles. For example, CM hAMSC is enriched by metalloproteinase (MMPs) inhibitors (TIMP1 and TIMP2) able to inhibit the MMPs aberrantly expressed in muscle biopsies from DMD patients and involved in disease progression [43]. CM hAMSC also contains Inhibin beta A chain (INHBA) that was found to stimulate proliferation of cardiomyocytes, leading to an accelerated cardiac recovery and scar clearance after injury [44]. Interestingly, CM hAMSC also contains Growth Differentiation Factor 15 (GDF15), a myomitokine released from skeletal muscle fibers with an important role in energy metabolism, whose role in muscle diseases is still controversial [45]. In addition, EVs from hAMSC carry some miRNAs with a critical role in regulating post-transcriptional expression of genes involved in skeletal muscle differentiation and regeneration. For example, miR-24-3p is present with a genetic weight of 17.73% in EVs from hAMSC and, by regulating high mobility group AT-hook 1 (HMGA1)/inhibitor of differentiation 3 (ID3) axis, has been shown to promote myoblast differentiation and skeletal muscle regeneration [46]. Other interesting miRs detected in EV hAMSC were reported to ensure proper myoblast differentiation into myotubes: miR-26a that targets the transcription factors Smad1 and Smad4, critical for the TGF-β/BMP pathway; and miR-29a-3p that targets the transcription factor YY1, Akt3, HDAC4, and miR-214-3p that targets N-ras [46,47,48,49].

It is also known that in skeletal muscle cells, miR-214 and miR-26a may target the Polycomb group (PcG) Ezh2 methyltransferase, a negative regulator of muscle differentiation, at distinct developmental myogenic stages. Upregulation of miR-26a is evident at the latest stages of myoblast cell differentiation and initial reduction of the Ezh2 protein level and concomitant recruitment of MyoD and myogenin at the *Dnm3* locus at the earlier differentiation step coincides with activation of miR-214, a microRNA that also targets Ezh2, supporting its own expression and cell differentiation [50,51,52,53,54,55,56,57].

Herein, we identified a remarkable role for the hAMSC secretome and in particular for EVs derived thereof to promote at the same time, the expansion of MuSCs and their differentiation toward functional muscle cells providing a valuable and clinically applicable pharmacological tool to regenerate diseased muscles, such as in muscular dystrophies. 

The treatment of DMD based on EV hAMSC that we have elucidated, although it does not cure the genetic defect, represents a therapeutic strategy to expand the regenerative window of dystrophic muscles, improving muscle regeneration, and delaying muscle degeneration. EV hAMSC administration could be of great importance to expand patient life-span and can be envisioned as combinatory treatment given that extension of the regenerative capacity of dystrophic muscle is functional for the effectiveness of other treatments, such as gene or cell therapy.

In light of the results obtained, new studies should be based on systemic injection of EV hAMSC sub-cutaneously or intravenously to extend the area of regenerative-action on all muscle tissues compromised by the genetic defect.

## 4. Materials and Methods

### 4.1. Isolation, Culture of hAMSC and Preparation of CM 

Human term placentae were collected at the Department of Obstetrics and Gynecology of Fondazione Poliambulanza hospital in Brescia from healthy women after vaginal delivery or caesarean section at term. Placentae were collected after obtaining informed written consent according to the guidelines set by the local ethics committee (Comitato Etico Provinciale of Brescia, Italy: number NP 2243, approved 19 January 2016). hAMSC were isolated from the amniotic membrane using well-established techniques [32]. Briefly, amniotic membrane was cut in fragments and enzymatically digested at 37 °C with 2.5 U/mL dispase (VWR, Radnor, PA, USA). The digestion continued in the presence of collagenase and DNase I (both from Roche, Basel, Switzerland). The supernatant of cell suspension obtained by low centrifugation was centrifuged at 300× *g* for 10 min to collect the cells. After isolation, hAMSC (p0) were phenotypically characterized as previously reported (30). Cells with >80% expression of mesenchymal markers CD13 and CD90 and <10% expression of the hematopoietic marker CD45 and of the epithelial marker CD324 were used in this study. Freshly isolated cells referred to as hAMSC p0 were expanded until passage 1 (p1) by plating at a density of [10^4^ cells/cm^2^] in Chang medium C (Irvine Scientific, Santa Ana, CA, USA) supplemented with 2 mM L-glutamine (Sigma-Aldrich, St. Louis, MO, USA) at 37 °C in 5% CO_2_. Conditioned medium from hAMSC was generated by culturing hAMSC p1 for 5 days in 24-well plates (Corning Inc., Corning, NY, USA) (0.5 × 10^6^ cells/well in a final volume of 0.5 mL), in serum-free Gibco™ DMEM/F-12, HEPES complete medium compose of DMEM/F-12, HEPES supplemented with 2 mM L-glutamine (Sigma-Aldrich) and 100 U/mL penicillin and 100 μg/mL streptomycin (herein referred to P/S, all from Merck, St. Louis, MO, USA). The control (CTR) was DMEM/F-12, HEPES complete medium that was left for 5 days at 37 °C in 5% CO_2_. After 5 days, the supernatants (or control media) were collected, centrifuged at 300× *g* for 10 min, filtered through a 0.2-μm sterile filter (Sartorius Stedim, Florence, Italy) and stored at −80 °C until use.

For the preparation of hAMSC-conditioned media devoid of EVs, during cell expansion to p1 hAMSC were treated, for 30 min with the neutral sphingomyelinase inhibitor GW4869 (10 µM) which selectively blocks exosome biogenesis or with respective vehicle (DMSO). Treated hAMSC were then washed, detached, and replated to produce CM as described above. 

### 4.2. EVs Isolation and Characterization

EVs were isolated from CM hAMSC and in parallel from CM CTR with Total Exosomes Isolation Reagent-TEIR (#4478359, Invitrogen by Thermo Fisher Scientific, Waltham, MA, USA) according to manufacturer instructions. 

#### 4.2.1. Field Emission Scanning Electron Microscopy

Ultrastructural analysis of EVs was performed by field emission scanning electron microscopy (FESEM). EVs suspension was allowed to dry on 13-mm-diameter glass coverslip. EVs were fixed with 2.5% *v*/*v* glutaraldehyde in 0.1 mol/L cacodylate buffer (pH 7.4) at room temperature for 1 h, washed in cacodylate buffer, and dehydrated through graded ethanols (30, 50, 70, 85, 95, 100%-10 min each). After 100% ethanol, 1:1 ethanol:hexamethyldisilazane (HMDS) was added for 3–5 min at RT followed by a final step of 3–5 min incubation in HMDS and air-drying. A glass coverslip was glued on the SEM pin stub and gold-coated (V150R quorum). Finally, EVs were examined by FESEM (Sigma-Zeiss). 

#### 4.2.2. Nanosight

The concentration and size of secreted vesicles was quantified by the nanoparticle tracking analysis (NTA) system (Nanosight NS300) and the number of particles isolated from 1 mL of CM was determined. 

#### 4.2.3. Western Blot

EVs that were isolated were lysed for protein extraction in RIPA buffer (50 mM Tris-HCl, pH 7.4; 150 mM NaCl; 1% NP-40; protease inhibitors). The total extravesicular protein content was quantified using the micro bicinchonic acid protein assay (BCA) (#23235, Thermo Fisher scientific). 

Western blot was performed on 12 µg of EV proteins using rabbit anti-CD81 (1/500, Thermo Fisher Scientific #PA513582) and mouse anti-Alix (3A9) (1/500, Cell Signaling Technologies (CST) #2171, Danvers, MA, USA). 

### 4.3. Experimental Model and Animal Procedure 

Animal procedures performed according to European Guidelines (2010/63/EU) and Italian law requirements (D.L. 26/2014) were approved by Animal Welfare Office, Department of Public Health and Veterinary, Nutrition and Food Safety, General Management of Animal Care, and Veterinary Drugs of Italian Ministry of Health and were, therefore, performed in accordance with the ethical standards laid down in the 1964 Declaration of Helsinki and its later amendments. *C57BL6*/*mdx* mice were bred, handled, and maintained under housing and feeding conditions according to the standard animal facility procedures and the ethics committee of the Fondazione Santa Lucia. Mice were euthanized by cervical dislocation. Efforts were made to minimize animal suffering and the number of animals necessary to produce reliable results. 

hAMSC-derived EVs at a final concentration of 3.2 × 10^9^ particles in 20 µL of PBS1x were injected 3 times in the left TA of 1.5 month-old *C57BL6*/*mdx* mice, every 7 days for 21 days. The rights TA were injected with vehicle (20 µL PBS1x) following the same timing described for EV injection. 

### 4.4. Human Myoblast Culture 

Human DMD myoblasts were kindly provided by Dr. Pier Lorenzo Puri, Rome, in collaboration with Telethon Biobank. Informed consent to use Telethon Network of Genetic Biobanks biological samples for diagnosis and research was obtained from the patients in accordance with protocols approved by the Institutional Review Board of the C. Besta Neurological Institute (Milan). Primary cultures were derived by enzymatic digestion of excised human skeletal obtain single cell. Cells were cultured in DMEM+glucose supplemented with 20% FBS (Corning Fetal Bovine Pen/Strep; 500 uL insulin (Sigma Aldrich #I9278); 10 ng/mL epidermal growth factor (EGF) (Thermo #PHG0311); and 1 ng/mL fibroblast growth factor (FGF) (PreproTech (Cranbury, NJ USA) #100-18B). 

### 4.5. Isolation and Culture of MuSCs 

Murine MuSCs were isolated from 1.5 month-old C56BL6/*mdx* tibialis anterior, gastrocnemius and extensor digitorum longus muscles following Mozzetta’s protocol [58]. Briefly, tissues were digested by Dispase II/Collagenase A and cells were isolated based on their size, granulosity, and fluorophore levels using a FACS MoFlo Astrios EQ High Speed Cell Sorter (Beckman Coulter, Brea, CA, USA) and analyzed using FlowJo. In detail, MuSCs were isolated as TER119neg/CD45neg/CD31neg/alpha7INTEGRINpos/SCA-1neg cells. MuSCs were cultured after sorting directly in growth culture media GM2: 20% FBS (#16000044, Gibco), 10% HS (#26050-070, Gibco), 1% penicillin–streptomycin (#15140, Gibco), 1% chicken embryo extract (CEE, Seralabs GENTAUR GmbH, Germany #CE-650-F) in DMEM + Pyruvate (Gibco ThermoFisher Scientific-US #41966). MuSCs were plated at low density on regular cell culture dishes coated with gelatin 0.1% (Stemcell Technologies USA #07903) and then treated with CM hAMSC and CM CTR diluted 1:5 in the culture medium or with 6.5 × 10^8^ EVs hAMSC isolated from the same amount of diluted CM hAMSC used for treatment. 

### 4.6. Single Fiber Isolation and Culture 

Single fibers were isolated from gastrocnemius, soleus, and extensor digitorum longus muscles of 1.5 month-old *C57BL6J-WT* mice. The muscles were digested at 37 °C under gentle agitation for 45 min in digestion solution (DMEM + pyruvate +4.5 g/L glucose + glutamate, 10 units/mL penicillin and 10 μg/mL streptomycin, 0.35% Collagenase I (#C0130, SIGMA). After serial washes to remove debris, the single fibers were cultured floating in a 100 mm Petri dish pre-coated with pre-warm proliferating medium (GM1: DMEM + pyruvate + 4.5 g/L glucose + glutamate, 10% horse serum, 0.5% chicken embryo extract) for 24 h. Then, the single fibers were gently and carefully moved into a low-adherence 6-well plate and exposed for the next 48 h to CM hAMSC or CM CTR diluted 1:5 in GM1. Subsequently, single fibers were fixed with 4% PFA for immunofluorescence analysis [58].

### 4.7. Immunofluorescence 

For immunofluorescence analysis, cryo-sections, and cells were fixed in 4% PFA for 10 min and permeabilized with 100% cold acetone (#32201, Sigma) for 6 min at −20 °C or 100% cold methanol (#32213, Sigma) for 6 min at −20° or with 0.25% Triton for 15 min at RT. Muscle sections were blocked for 1 h with a solution containing 4% BSA (#A7030, Sigma) in PBS. The primary antibodies incubation was performed O.N. at 4 °C and then the antibody binding specificity was revealed using secondary antibodies coupled to Alexa Fluor 488, 594, or 647 (Invitrogen). Sections were incubated with DAPI in PBS for 5 min for nuclear staining, washed in PBS, and mounted with glycerol 3:1 in PBS. The primary antibodies used for immunofluorescences were: rabbit anti-laminin (1/400, #L9393, Sigma); mouse anti-eMyHC (1:20, #F1.652, Developmental Studies Hybridoma Bank, DSHB, University of Iowa, USA); mouse anti-MF20 (1:20, Developmental Studies Hybridoma Bank, DSHB, University of Iowa, USA), mouse anti-PAX7 (1:10, Developmental Studies Hybridoma Bank, DSHB, University of Iowa, USA), rabbit anti-MyoD-318 (1:50 #SC760, Santa Cruz Biotechnology, Dallas, TX, USA), EDU (#C10350, Invitrogen), rabbit anti-Ki67 (1:1000, #15580 Abcam). Myoblast fusion, essential for muscle regeneration, was evaluated by the fusion index analysis. Specifically, MuSCs were stained with anti-MF20 to detect MyHC and the percentage of nuclei were measured, and more specifically the nuclei that were (i) in MyHC- or MyHC+ mononucleated myotubes (n < 2), (ii) inside myotubes containing between two and five nuclei (2 < n < 5), (iii) inside myotubes containing more than five nuclei (n > 5). Collagen deposition was evaluated through Sirius Red staining. Muscle cryosections were fixed for 1 h at 56 °C in Bouin’s solution and then stained in Picro-sirius red (0.1%) solution for 1 h (Direct Red 80; #365548-5G Sigma and picric acid solution; #P6744-1GA Sigma). Then, the sections were washed in acidified water 0.5% *v*/*v* and fixed in 100% ethanol. The final dehydration was performed in xylene 100% and sections were mounted with Eukitt (#03989 Sigma). 

Muscle cryosections were fixed for 5 min at room temperature with 2% PFA and washed three times with PBS. Picro-sirius red staining (Direct Red 80 (Sigma #365548-5G)) in picric acid solution (Sigma #P6744-1GA)). Tissue area positive for Picro-sirius red staining was quantified by image analysis (Image J 1.44) [59]. 

### 4.8. RNA Preparation and RT-qPCR

TRIzol Reagent (#T9424, Sigma) was used to extract total RNA, including small RNA, according to the manufacturer’s recommendations and 0.5–1 μg was retro-transcribed using the TaqMan reverse transcription kit (Applied Biosystems, Waltham, MA, USA). Real-time qPCR was performed using TB Green *Premix Ex Taq* II (Tli RNase H Plus, Takara, Kusatsu, Japan) that includes TB Green, a reagent designed for intercalator-based qPCR and the following primers: 

MmCol3a1 Fw: 5′ CCCAACCCAGAGATCCCATT 3′; MmCol3a1 Rev: 5′ GGTCACCATTTCTCCCAGGA 3′; MmCol1a1 Fw: 5′ CCTCAGGGTATTGCTGGACA 3′; MmCol1a1 Rev: 5′ GAAGGACCTTGTTTGCCAGG 3′; MmPax7 FW: 5′ AGGACGACGAGGAAGGAGA 3′; MmPax7Rev: 5′ TCATCCAGACGGTTCCCTTT 3′; MmKi67 FW: 5′ TCACCTGGTCACCATCAAGC 3′; MmKi67 Rev: 5′ TCAATACTCCTTCCAAACA 3′; MmMyoG FW 5′ GTCCCAACCCAGGAGATCA; MmMyoG Rev: 5′ CAGACATATCCTCCACCGT 3′. 

### 4.9. Statistical Analysis 

The number of independent experiments, replicates, and precision measures are reported in the figure legends (n, mean ± SEM). Statistical analysis was performed using Prism 8.2.1 software (Graph Pad Prism Software). Unpaired *t*-tests were used for comparing two different groups; one-way analysis of variance (one-way ANOVA) was used to analyze differences between more than two different groups (i.e., count of the mean of nuclei), while two-way analysis of variance (two-way ANOVA) was used for comparing different groups to examine the influence of different independent variables on dependent variables (i.e., fusion index or Pax7 and Myod quantification). When data met the assumptions of the tests (e.g., normal distribution) and the ANOVA was significant, post hoc testing of differences between groups were performed using Tukey’s honestly significant difference (HSD). When data did not meet the assumptions of the tests (e.g., normal distribution) but the ANOVA was significant, post hoc testing of differences between groups was performed using Kruskal–Wallis test. A *p* < 0.05 was considered statistically significant. No statistical method was used to predetermine sample size for animal studies. The animal experiments were not randomized. The investigators were not blinded to allocation during experiments and outcome assessment. No exclusion criteria were applied to exclude samples or animals from analysis. 

## 5. Conclusions

Here, for the first time, we demonstrated the beneficial effect of the hAMSC secretome in Duchenne muscular dystrophy.

In particular, we dissected the effect of hAMSC-secreted bioactive molecules by evaluating, in vitro, the activity of both the whole secretome (CM hAMSC) and its derived extracellular vesicles (EV hAMSC). We found that both CM hAMSC and EV hAMSC improve the proliferation and differentiation potential of human myoblast and mouse dystrophic muscle stem cells (MuSCs). Interestingly, we observed that the ability of muscle stem cells to differentiate is mainly enhanced by the co-culture with the EV hAMSC counterpart.

Finally, through in vivo experiments, we validated the regenerative potential of EV hAMSC and studied their effect in the mouse model of DMD-*mdx* mice. Intra-muscular treatment of young *mdx* mice with EVs released from hAMSC resulted in an increase of muscle regeneration and a decrease of fibrotic tissue deposition, counteracting the normal decline of dystrophic muscle. Interestingly, we also demonstrated the ability of EV hAMSC to act on the MuSCs niche compartment by promoting cell expansion. All our results here shown have been obtained by different experiments on a DMD animal model. All of them confirm the direct effects of hAMSCs-EVs on dystrophic MuSCs by activation of myogenic program; further studies will be necessary to fill the gap about their mechanism.

The future prospective of this study is to develop a new powerful pharmacological treatment based on using EV hAMSC to regenerate diseased muscles, preventing systemic side effects of cell therapies.

## Data Availability

The data generated or analyzed during the current study are available from the corresponding author on reasonable request.

## Supplementary Materials

The following supporting information can be downloaded at: https://www.mdpi.com/article/10.3390/ijms241512457/s1.
