# Supplementary material for: Amniotic Membrane-Derived Stromal Cells Release Extracellular Vesicles That Favor Regeneration of Dystrophic Skeletal Muscles
Source: Int J Mol Sci. 2023 Aug 5;24(15):12457. doi: 10.3390/ijms241512457 (PMC10418925; doi:10.3390/ijms241512457)
Supplement: Supplementary file 1 [file ijms-24-12457-s001.zip › ijms-2525385-supplementary.pdf]

Figure S1

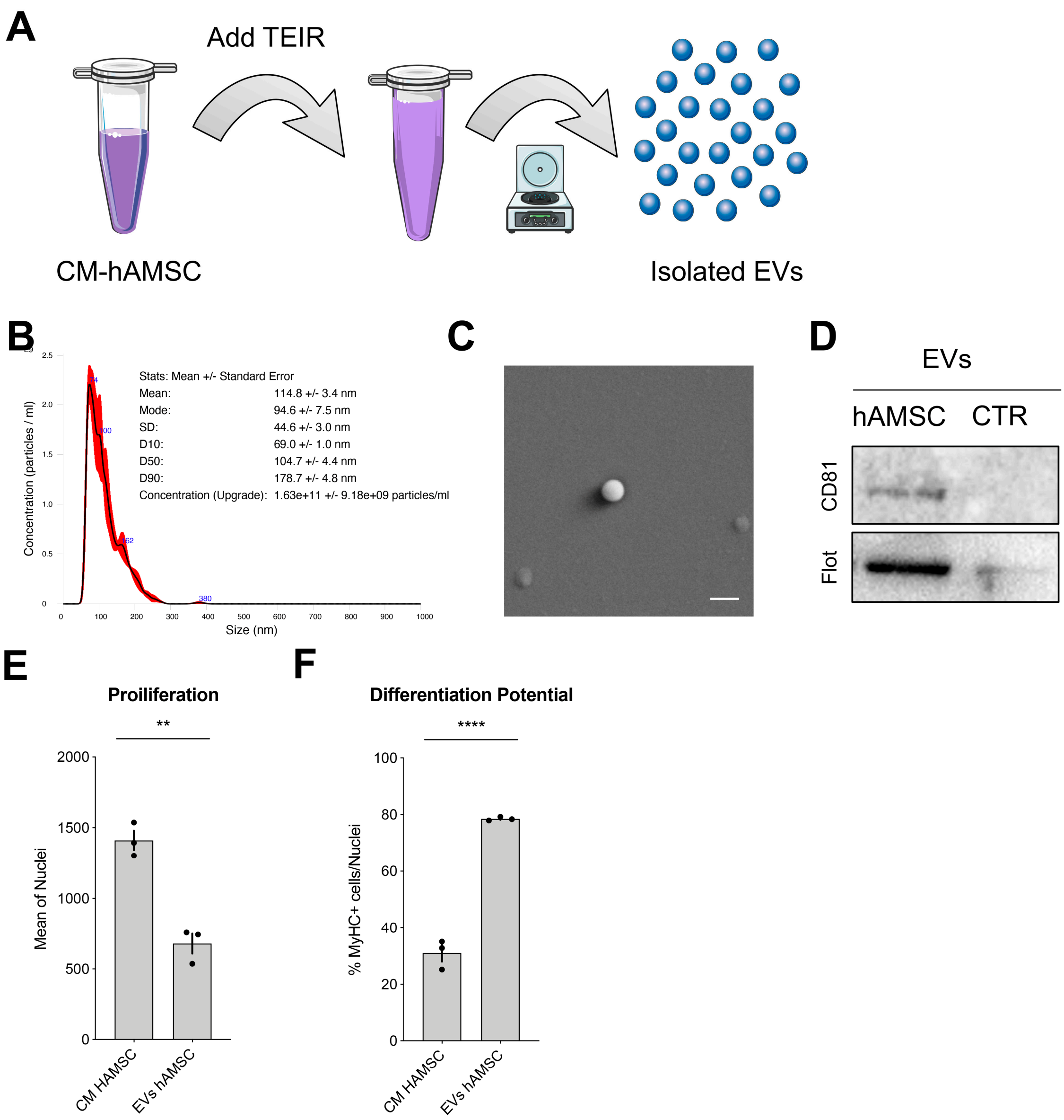

Figure S1. EVs hAMSC display physical features of exosomes

A) Representative scheme of EVs isolation from serum-free Conditioned Media of hAMSC using the Total Isolation Reagent (TEIR).

B) Nanoparticle tracking analysis (NTA) of the EVs isolated from serum-free Conditioned Media of hAMSC. The Line graphs corresponding to average concentration (particles/mL) and size (nm) of particles isolated.

C) Representative FESEM micrograph of EVs hAMSC obtained at an accelerating voltage of 4 kV (50,000× magnification). Scale bar = 200 µm.

D) Western blot analysis for CD81 and ALIX antibodies in EVs from serum-free Conditioned Media of hAMSC (EVs hAMSC) and CM CTR (EVs CTR). Full-length blots are presented in Figure S3.

E) Graphs showing the mean of nuclei number in MuSCs cultured with Conditioned Media of hAMSC (CM hAMSC) and EVs from serum-free Conditioned Media of hAMSC (EVs hAMSC). (n = 3, biological replicates). All data correspond to the average ± SEM. Asterisk (*) indicates statistical analysis by unpaired t-test; ** $p < 0.01$.

F) Graphs showing the percentage of MyHC positive cells in the condition described in e. (n = 3, biological replicates). All data correspond to the average ± SEM. Asterisk (*) indicates statistical analysis by unpaired t-test; **** $p < 0.0001$.

Figure S2

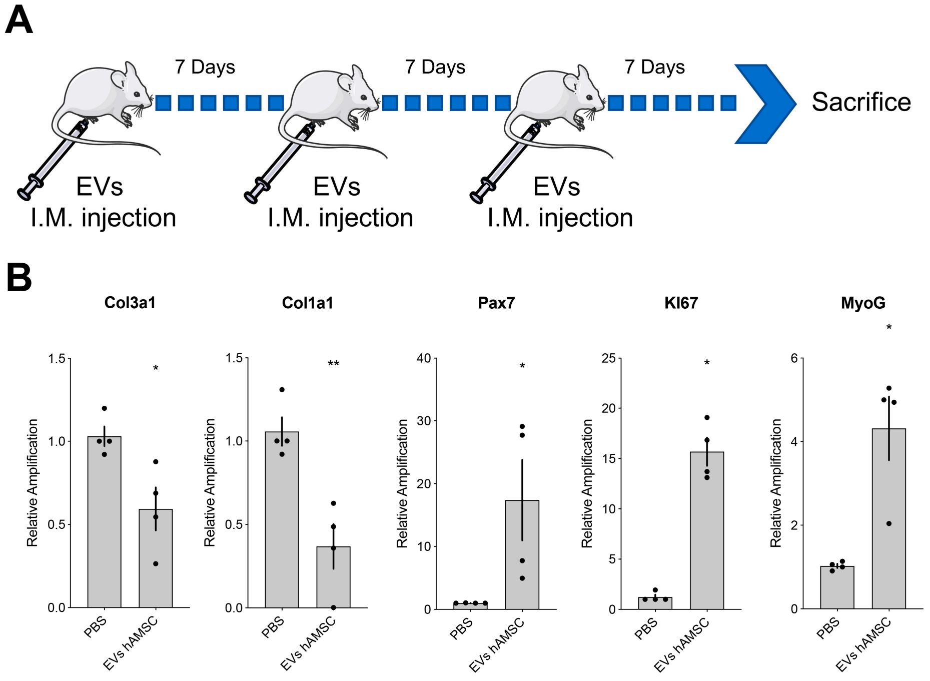

Figure S2. Beneficial effect of intramuscularly injected EVs hAMSC.

A) Representative scheme of EVs transplantation in mdx mice. Isolated EVs from hAMSC (EVs hAMSC) or PBS were injected in tibialis anterior of mdx mice every 7 days for 21 days.

B) Graphs showing the expression levels of several genes in muscle of PBS or EVs hAMSC treated mdx mice. Asterisk (*) indicates statistical analysis by unpaired *t*-test; * $p < 0.05$, ** $p < 0.01$. All data correspond to the average ± SEM. (n = 4, biological replicates).
